# The benefit and risk of PD-1/PD-L1 inhibitors plus anti-angiogenic agents as second or later-line treatment for patients with advanced non-small-cell lung cancer: a systematic review and single-arm meta-analysis of prospective clinical trials

**DOI:** 10.3389/fimmu.2023.1218258

**Published:** 2023-08-08

**Authors:** Shubin Chen, Wanying Mo, Wei Jiang, Shaozhang Zhou, Haijie Gan, Qitao Yu

**Affiliations:** Medical Oncology Of Respiratory, Guangxi Medical University Cancer Hospital, Nanning, Guangxi, China

**Keywords:** advanced non-small cell lung cancer, second or later-line therapy, PD-1/PD-L1 inhibitors, anti-angiogenic agents, meta-analysis

## Abstract

**Background:**

Previous studies revealed that Programmed cell death protein 1 (PD-1)/Programmed cell death-Ligand protein 1 (PD-L1) inhibitors plus anti-angiogenic agents had extensive anti-tumor activities. However, almost all studies on the efficacy and safety of PD-1/PD-L1 inhibitors plus anti-angiogenic agents as second or later-line treatment for patients with advanced non-small cell lung cancer are non-randomized controlled trials with small sample sizes, which might lead to a lack of effective metrics to assess the effectiveness and safety of the therapeutic regimen. Here, this meta-analysis aimed to evaluate the efficacy and safety of PD-1/PD-L1 inhibitors plus anti-angiogenic agents as second or later-line treatment for patients with advanced non-small cell lung cancer

**Methods:**

A single-arm meta-analysis was performed, and published literature from PubMed, Web of Science and Embase databases as of January 13, 2023, was systematically retrieved. We used the Cochrane risk of bias tool and methodological index for non-randomized studies (MINORS) Methodological items to evaluate the quality of eligible clinical trials. Outcomes including overall response rate (ORR), disease control rate (DCR), progression-free survival (PFS), overall survival (OS), and adverse events (AEs) were extracted for further analysis. The random effect model is used to calculate the pooled parameters.

**Results:**

19 studies (16 were non-comparative single-arm clinical trials and 3 were randomized controlled trials) were enrolled in this meta-analysis. In terms of tumor response, the pooled ORR and DCR were 22.4% (95% CI, 16.6-28.1%) and 76.8% (95% CI, 72.6-81.1%), respectively. With regard to survival analysis, the pooled PFS and OS were 5.20 (95% CI, 4.46-5.93) months and 14.09 (95% CI, 13.20-14.97) months, respectively. The pooled grade ≥3 adverse effect (AE) rate was 47.6% (95% CI, 33.1-62.0%)

**Conclusion:**

PD-1/PD-L1 inhibitors plus anti-angiogenic agents has promising efficacy and safety as second or later-line treatment in patients with advanced non-small cell lung cancer.

**Systematic review registration:**

https://www.crd.york.ac.uk/prospero/, identifier CRD42023407559.

## Introduction

Non-small-cell lung cancer (NSCLC), which makes up around 80–85% of each diagnosis, is the most common kind of lung cancer and the leading cause of cancer-related death globally. The most recent “Global Cancer Statistics 2020” data from the World Health Organization show that its incidence rate is more than 1/10 of the world’s malignant tumors (1). Approximately 70% of patients with stage I to stage III non-small cell lung cancer are surgically curable (2). Only 5% of patients with advanced non-small cell lung cancer survived 5 years (3). About 62 percent of non-small cell lung cancer patients are given a stage IV diagnosis at their initial diagnosis because they don’t exhibit the typical signs of lung cancer (4). Chemotherapy, targeted therapy, and immunotherapy are the three most widely used therapies for persons with advanced non-small cell lung cancer (5).

Since the introduction of immunotherapy, the area of therapeutic approaches for NSCLC has taken on a whole new perspective and demonstrated considerable promise. Jianwei Zhu presented a review (6) of immunotherapy, which found that immunotherapies other than immune checkpoint inhibitor therapy, the current literature does not provide evidence that suggests a survival benefit from adding immunotherapy (excluding checkpoint inhibitors) to conventional curative surgery or radiotherapy, for people with localized NSCLC (stages I to III). However, patients with metastatic NSCLC who receive treatment with checkpoint inhibitors that target programmed cell death protein 1 (PD-1) and programmed death ligand-1 (PD-L1) have shown improved disease response rates and longer lifetime (7–9). The benefit of PD-1/PD-L1 inhibitor monotherapy is limited, a systematic review (10) was conducted by Fausto Petrelli et al. The study showed that the addition of immune checkpoint inhibitors to chemotherapy may improve both OS compared with chemotherapy alone, it is critically necessary to investigate the efficacy of combination treatment modalities to help doctors optimize their treatment regimens.

Anti-angiogenic agents, including monoclonal antibodies like bevacizumab and small molecule inhibitors like anlotinib, apatinib, and lenvatinib, inhibit the VEGF signaling pathway, exhibiting anti-tumor effects. Clinical studies have demonstrated the efficacy of this family of drugs in the treatment of advanced non-small cell lung cancer, including BEYOND (11) and ALTER0303 (12). Results from several clinical trials have been reported on the potential efficacy of PD-1/PD-L1 inhibitors in combination with anti-angiogenic drugs. Atezolizumab was shown to be beneficial in prolonging progression-free survival and overall survival in patients with advanced non-small cell lung cancer in IMpower150 (13) when used in conjunction with chemotherapy and bevacizumab. Pembrolizumab with lenvatinib was shown to have an objective response rate (ORR) of 33.3% when used as the first-line therapy for advanced non-small cell lung cancer in KEYNOTE-524 (14). These findings offer a scientific rationale for the PD-1/PD-L1 inhibitor plus anti-angiogenic drug therapy regimen.

Although various effective compounds for the second or later-line treatment of advanced non-small cell lung cancer improved the overall survival, the optimal regimen remains controversial. Due to the paucity of scientific evidence supporting the use of PD-1/PD-L1 inhibitors in combination with other drugs in the second or later-line treatment of patients with advanced non-small cell lung cancer, a number of clinical studies are being conducted worldwide to further examine the viability of combination regimens. However, almost all studies on the efficacy and safety of PD-1/PD-L1 inhibitors plus anti-angiogenic agents as second or later-line treatment for patients with advanced non-small cell lung cancer are non-randomized controlled trials with small sample sizes, which might lead to a lack of effective metrics to assess the effectiveness and safety of the therapeutic regimen. Here, this meta-analysis aimed to evaluate the efficacy and safety of PD-1/PD-L1 inhibitors plus anti-angiogenic agents as second or later-line treatment for patients with advanced non-small cell lung cancer

## Materials and methods

Single-arm meta-analysis is in accordance with PRISMA(Preferred Reporting Items for Systematic Review and Meta-Analysis) guidelines (15) and has been registered with PROSPERO (ID: CRD42023407559, https://www.crd.york.ac.uk/prospero/).

### Search strategy

We systematically searched PubMed, Embase and Web of Science databases as of January 13, 2023, for non-comparative clinical trials and randomized controlled trials (RCTs). The complete search we used for PubMed was supplied in **Table S1**. We also manually searched the abstract of European Society for Medical Oncology (ESMO) and American Society of Clinical Oncology (ASCO) for further eligible articles.

### Selection criteria

Studies that satisfied the following inclusion criteria were taken into consideration: (1) Patients in prospective clinical trials having an advanced NSCLC diagnosis confirmed by histology; (2) taking PD-1/PD-L1 inhibitors plus anti-angiogenic drugs as a second or later-line therapy; (3) Clinical tumor outcomes, such as the objective response rate (ORR), disease control rate (DCR), progression-free survival (PFS), overall survival (OS), and adverse events (AEs), were reported for patients. To reduce the possibility of bias, the following studies were disregarded: (1) Studies that did not cover NSCLC; (2) No prospective clinical trials; (3) Lack of essential data or overlapping studies; (4) animal experiments, cell research, reviews, meta-analyses, duplicates, case reports, or letters were not taken into consideration; Through inclusion and exclusion criteria, two scientists independently selected possible suitable articles. Any disagreements about the inclusion of the study were settled by these two or a third investigator.

### Data extraction and quality assessment

Two investigators independently retrieved the necessary data from each included studies after which the studies’ quality was evaluated. First author, publication year, registration number, country, patient count, histology, median age, proportion of men, trial phase, and trial design are a brief summary of the retrieved characteristics. Grade 3 AE, ORR, DCR, median PFS, and OS were among the outcomes that were also retrieved. We used the MINORS Methodological items (16) to assess the quality of non-comparative single-arm clinical trials, and the Cochrane risk of bias instrument (17) to assess the quality of eligible RCTs.

### Statistical analysis

Utilizing Stata statistical software, evidence synthesis was carried out. With Stata (Stata Corp, USA), we entered the overall clinical setting percentage for the primary outcome and the total number of research participants, and then computed the relevant standard errors of these quasinormal distribution “rates” using Stata. The lower interval (LI) and upper interval (UI), which have a 95% confidence level, may be justified using the “rates” and standard errors. Finally, the output included the pooled effect sizes (ES), which represented median “rates” and 95% confidence intervals (95% CI). The I2 statistic was used to analyze heterogeneity between studies. Studies were categorized as having low, moderate, or high heterogeneity based on their I2 statistics, which ranged from 25 to 50%, 50 to 75%, and >75%. For the I2 test, substantial heterogeneity was defined as P 0.05. We used random-effects models for all pooled ES because there was great subjectivity given the lack of related control groups in the noncomparative studies, and a tendency toward high heterogeneity. The large amount of data that was provided allowed for the meta-regression and subgroup analyses to be carried out. Additionally, sensitivity analysis was done to assess the consistency and dependability of the information that was merged. Finally, Egger’s tests looked for a possible publication bias.

## Results

### Study identification

**Figure 1:**
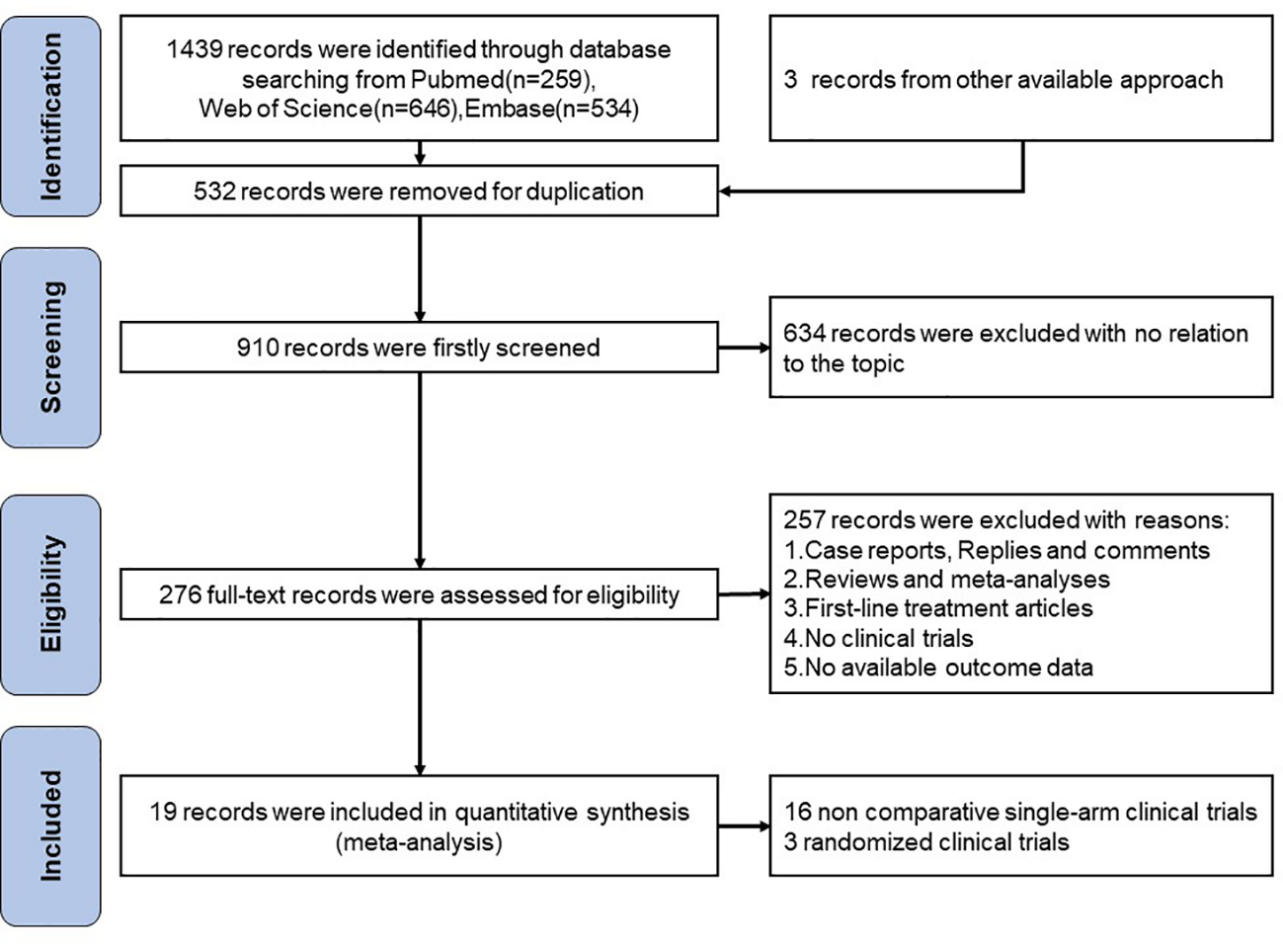
Flow chart of the single-arm meta-analysis.

A total of 1439 records were found after searching the aforementioned databases Pubmed (n=259), Web of Science (n=646) and Embase (n=534), we also found 3 more records in the abstracts of the ESMO and ASCO conferences. We excluded 532 articles for duplication. 634 records were excluded with no relation to the topic. 257 of records excluded with reasons:(1) Case reports, Replies and comments;(2)Reviews and meta-analyses;(3)First-line treatment articles;(4)No clinical trials;(5)No available outcome data. For the remaining 19 publications, a quantitative synthesis was possible. The selection procedure was depicted in **Figure 1**.

### Study characteristics

The present single-arm meta-analysis included a total of 19 studies (18–36) involving 931 participants; **Table 1** describes the main study characteristics, and **Table 2** presents the outcome results. The studies were all published between 2019 and 2022. Three studies (25, 28, 30) were randomized clinical trials, and 16 studies (18–24, 26, 27, 29, 31–36) were non-comparative clinical trials. Nine studies (19, 22, 23, 25–27, 30, 34, 35) were published abstracts, and 10 studies (18, 20, 21, 24, 28, 29, 31–33, 36) were original studies.

**Table 1:** Characteristics of clinical trials included in the single-arm meta-analysis.

Study	Year	Register number	Country	Patients	Histology	Age (years)	Male (%)	Phase	Design
Herbst et al. (18)	2019	NCT02443324	5-countries^*^	27	NSCLC	65.0	78.0%	Ia/Ib	Single-arm
Galffy et al. (19)	2020	NCT03472560	Hungary	41	NSCLC	NR	NR	II	Single-arm
Bang et al. (20)	2020	NCT02572687	8-countries^^^	28	NSCLC	64.5	68.0%	Ia/Ib	Single-arm
Zhou et al. (21)	2021	NCT04670107	China	45	NSCLC	62.0	72.5%	Ib	Single-arm
Puri et al. (22)	2021	NCT03377023	USA	18	NSCLC	NR	66.7%	Ib/II	Single-arm
Pan et al. (23)	2021	ChiCTR2000034597	China	10	NSCLC	NR	33.3%	II	Single-arm
Zhou et al. (24)	2021	NCT04203485	China	105	N-Sq NSCLC	58.0	75.2%	Ib/II	Single-arm
Han et al. (25)	2021	NCT03910127	China	68	NSCLC	NR	NR	II	RCT
Leal et al. (26)	2022	NCT02954991	USA	68	N-Sq NSCLC	66.0	43.0%	II	Single-arm
Fang et al. (27)	2022	NCT04426825	China	19	EGFR+NSCLC	63.0	42.0%	II	Single-arm
Reckmap et al. (28)	2022	NCT03971474	USA	69	NSCLC	66.4	59.0%	II	RCT
Lv et al. (29)	2022	ChiCTR1900023664	China	34	NSCLC	60.0	67.6%	II	Single-arm
Lu et al. (30)	2022	NCT03802240	China	158	EGFR+ NSCLC	58.5	41.1%	III	RCT
Lee et al. (31)	2022	NCT03616691	Korea	24	NSCLC	63.0	54.2%	II	Single-arm
Herzog et al. (32)	2022	NCT03689855	USA	21	NSCLC	67.0	19.0%	II	Single-arm
Gao et al. (33)	2022	NCT03083041	China	25	Sq NSCLC	63.0	92.0%	II	Single-arm
Neal et al. (34)	2022	NCT03170960	9-countries^#^	81	N-Sq NSCLC	67.0	57.0%	Ib	Single-arm
Gao et al. (35)	2022	NCT03666143	China	47	NSCLC	60.0	NR	Ib	Single-arm
Gao et al. (36)	2022	NCT03083041	China	43	EGFR/ALK+ NSCLC	55.0	58.1%	II	Single-arm

5-countries^*^: USA, France, Germany, Spain and the UK; 8-countries^^^: France, Germany, Israel, Italy, Republic of Korea, Spain, Taiwan, and the USA; 9-countries^#^: Australia, Belgium, France, Germany, Italy, Netherlands, Spain, United Kingdom and the USA.

**Table 2:** Original data extracted from included clinical trials.

Study	Year	Patients	Intervention	DCR(%)	ORR(%)	mPFS(months)	mOS(months)	Grade 3-4 AE(%)
Herbst et al. (18)	2019	27	Pembrolizumab Ramucirumab	85.0%	30.0%	9.7(4.6-27.6)	26.2(11.8-NR)	NR
Galffy et al. (19)	2020	41	Avelumab Axitinib	70.7%	31.7%	5.5(2.5-7.0)	NR	58.5%
Bang et al. (20)	2020	28	Durvalumab Ramucirumab	57.0%	11.0%	2.7(1.6-5.8)	11.0(6.2-15.2)	32.1%
Zhou et al. (21)	2021	45	Camrelizumab Anlotinib	82.2%	13.3%	8.2(4.3-12.1)	12.7(10.2-15.1)	03.7%
Puri et al. (22)	2021	18	Nvolumab Ipilimumab Nintedanib	61.0%	22.0%	2.7(1.4-NR)	7.7(5.0-NR)	NR
Pan et al. (23)	2021	10	Camrelizumab Chemotherapy Apatinib	80.0%	20.0%	NR	NR	NR
Zhou et al. (24)	2021	105	Camrelizumab Apatinib	73.3%	27.6%	5.7(4.5-8.8)	15.5(10.9-24.5)	69.5%
Han et al. (25)	2021	68	TQB-2450(PD-L1) Anlotinib	73.5%	30.9%	6.9(5.3-12.4)	NR	67.7%
Leal et al. (26)	2021	68	Nivolumab Sitravatinib	NR	16.0%	6.0	15(9.3-21.1)	60.0%
Fang et al. (27)	2022	19	Atezolizumab Bevacizumab	68.4%	15.8%	2.8	NR	40.0%
Reckmap et al. (28)	2022	69	Pembrolizumab Ramucirumab	75.0%	22.0%	4.5(4.2-6.1)	14.5(13.9-16.1)	42.0%
Lv et al. (29)	2022	34	Nivolumab Recombinant human endostatin	64.7%	41.2%	6.8(1.1-12.1)	17.1(6.6-27.6)	11.8%
Lu et al. (30)	2022	158	Sintilimab Bevacizumab biosimilar IBI305 Chemotherapy	86.1%	48.1%	7.2(6.6-9.3)	NR	59.5%
Lee et al. (31)	2022	24	Atezolizumab Bevacizumab	87.5%	12.5%	5.6(4.1-7.1)	14.0(10.7-17.4)	4.2%
Herzog et al. (32)	2022	21	Atezolizumab Ramucirumab	81.0%	4.80%	3.4	16.5	43.0%
Gao et al. (33)	2022	25	Camrelizumab Apatinib	84.0%	32.0%	6.0(3.5-8.1)	13.3(6.4-18.8)	84.0%
Neal et al. (34)	2022	81	Atezolizumab Cabozantinib	80%	19.0%	4.5(3.5-5.6)	13.8(7.2-15.7)	52.0%
Gao et al. (35)	2021	47	Tislelizumab Sitravatinib	86.0%	14.0%	5.2(4.1-5.9)	NR	68.0%
Gao et al. (36)	2022	43	Camrelizumab Apatinib	58.1%	18.6%	2.8(1.9-5.5)	NR	65.1%

### Quality assessment

Utilizing the Cochrane risk of bias tool, the three RCTs (25, 28, 30) were evaluated and did not demonstrate allocation concealment, but generated random sequences, provided complete outcome data, reported no selective outcome, and were free of other bias (**Figure S1**). To evaluate the non-comparative single-arm clinical trials’ quality, we utilized the MINORS Methodological items; the quality evaluation specifics are included in **Table 3**.

**Table 3:** Quality assessment of the non-comparative single-arm clinical trials included in the meta-analysis.

Study	Q1	Q2	Q3	Q4	Q5	Q6	Q7	Q8	Score†
Herbst et al. (18)	2	2	2	2	0	2	2	2	14
Galffy et al. (19)	2	2	2	2	0	2	0	2	12
Bang et al. (20)	2	2	2	2	0	2	2	2	14
Zhou et al. (21)	2	2	2	2	0	2	2	2	14
Puri et al. (22)	2	2	2	2	0	2	2	1	13
Pan et al. (23)	2	2	2	2	0	2	1	0	11
Zhou et al. (24)	2	2	2	2	0	2	1	2	13
Leal et al. (26)	2	2	2	2	0	2	2	1	13
Fang et al. (27)	2	2	2	2	0	2	2	0	12
Lv et al. (29)	2	2	2	2	0	2	1	2	13
Lee et al. (31)	2	2	2	2	0	2	2	2	14
Herzog et al. (32)	2	2	2	2	0	2	2	1	13
Gao et al. (33)	2	2	2	2	0	2	2	2	14
Neal et al. (34)	2	2	2	2	0	2	2	2	14
Gao et al. (35)	2	2	2	2	0	2	2	2	14
Gao et al. (36)	2	2	2	2	0	2	2	2	14

Numbers Q1-Q8 in heading signified:

Q1: A clearly stated aim: the question addressed should be precise and relevant in the light of available literature.

Q2: Inclusion of consecutive patients: all patients potentially fit for inclusion (satisfying the criteria for inclusion) have been included in the study during the study period (no exclusion or details about the reasons for exclusion).

Q3: Prospective collection of data: data were collected according to a protocol established before the beginning of the study.

Q4: Endpoints appropriate to the aim of the study: unambiguous explanation of the criteria used to evaluate the main outcome which should be in accordance with the question addressed by the study. Also, the endpoints should be assessed on an intention-to-treat basis.

Q5: Unbiased assessment of the study endpoint: blind evaluation of objective endpoints and double-blind evaluation of subjective endpoints. Otherwise the reasons for not blinding should be stated.

Q6: Follow-up period appropriate to the aim of the study: the follow-up should be sufficiently long to allow the assessment of the main endpoint and possible adverse events.

Q7: Loss to follow up less than 5%: all patients should be included in the follow up. Otherwise, the proportion lost to follow up should not exceed the proportion experiencing the major endpoint.

Q8: Prospective calculation of the study size: information of the size of detectable difference of interest with a calculation of 95% confidence interval, according to the expected incidence of the outcome event, and information about the level for statistical significance and estimates of power when comparing the outcomes.

†The items are scored 0 (not reported), 1 (reported but inadequate) or 2 (reported and adequate).

### Therapeutic efficacy assessments of ORR

**Figure 2:**
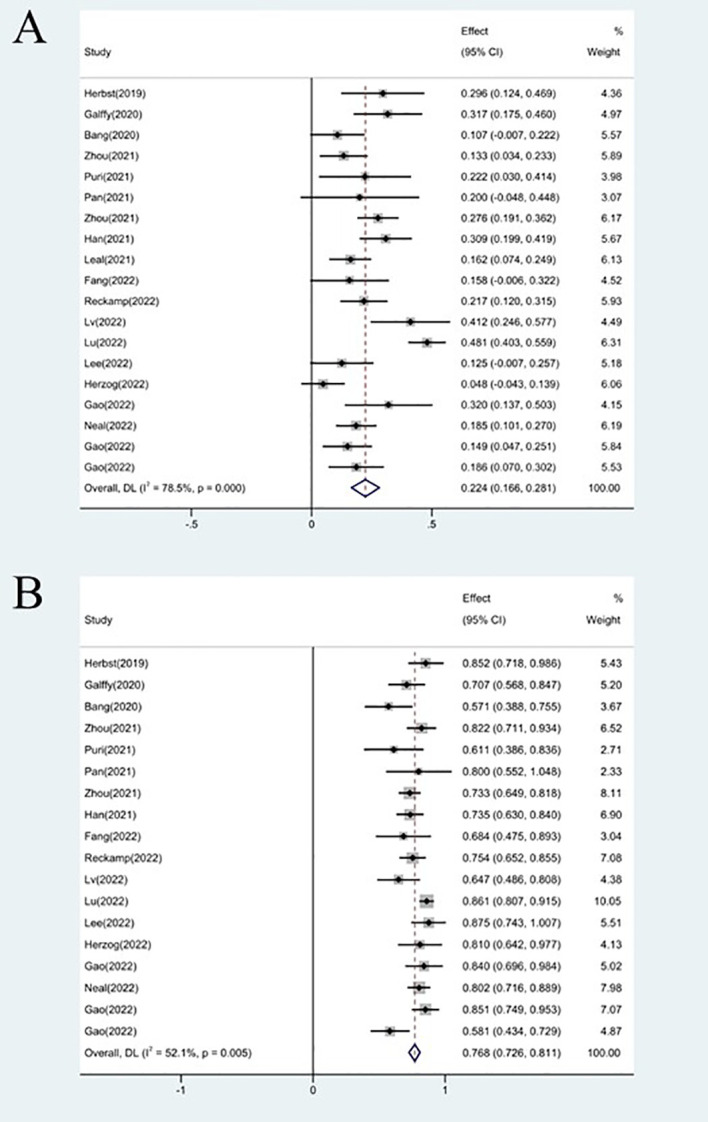
Forest plot about the pooled results of ORR **(A)** and DCR **(B)**.

**Figure 3:**
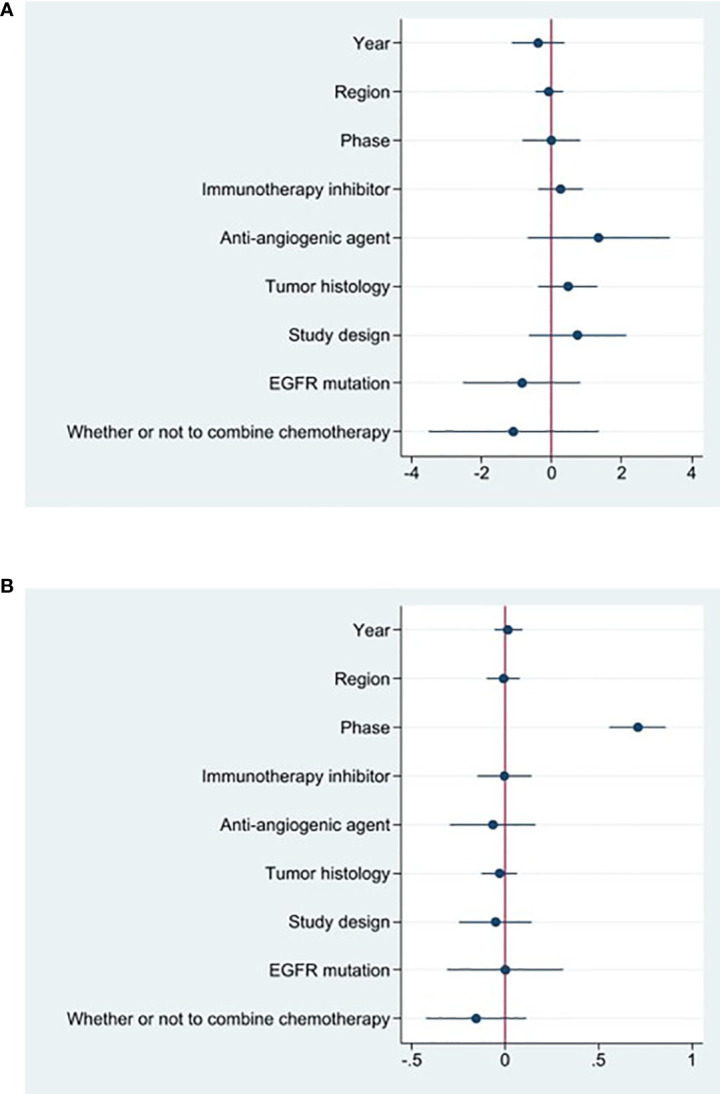
Coefplot of the results of meta-regression of ORR **(A)** and DCR **(B)**.

**Figure 4:**
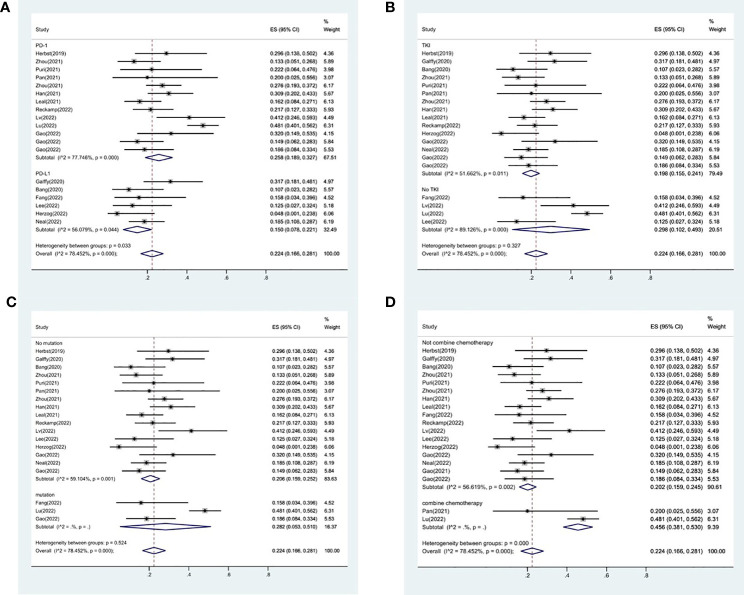
Subgroup analysis of ORR in immunotherapy inhibitor **(A)**, anti-angiogenic agent **(B)**, EGFR mutation **(C)** and whether or not to combine chemotherapy **(D)**.

The effectiveness response was recorded in every study analyzed. The ORRs ranged from 11 to 48% among the investigations. The analysis revealed considerable heterogeneity (I^2 =^ 78.5%, P<0.0001) and a pooled ORR of 22.4% (95% CI: 16.6%-28.1%, **Figure 2A** **)**. We further evaluated possible sources of heterogeneity by using meta-regression because there was high ORR heterogeneity across trials. For the meta-regression analysis, we selected 9 variables (year, region, phase, immunotherapy inhibitor, anti-angiogenic drug, tumor histology, study design, EGFR mutation and whether or not to combine chemotherapy). According to the meta-regression findings, there is no statistically significant difference between the p-values for each variable **(** **Figure 3A** **)**. The results failed to clearly identify significant influences on heterogeneity. We selected four variables(immunotherapy inhibitor, anti-angiogenic agent, EGFR mutation and whether or not to combine chemotherapy) for further subgroup analysis. Subgroup analysis revealed that the pooled ORR in patients who received PD-1 as immunotherapy was 25.8% (95% CI: 18.9%–32.7%), which was higher than that of patients receiving PD-L1 for treatment, with a statistical difference. The pooled ORR of patients receiving TKI as treatment was 19.8% (95% CI: 15.5%–24.1%). The pooled ORR of patients without EGFR mutation was 20.6% (95% CI: 15.6%–25.2%). The pooled ORR of patients receiving chemotherapy was 45.6% (95% CI: 38.1%–53.0%), which was higher than that of patients who did not receive chemotherapy, with a statistical difference (**Figure 4**).

### Therapeutic efficacy assessments of DCR

**Figure 5:**
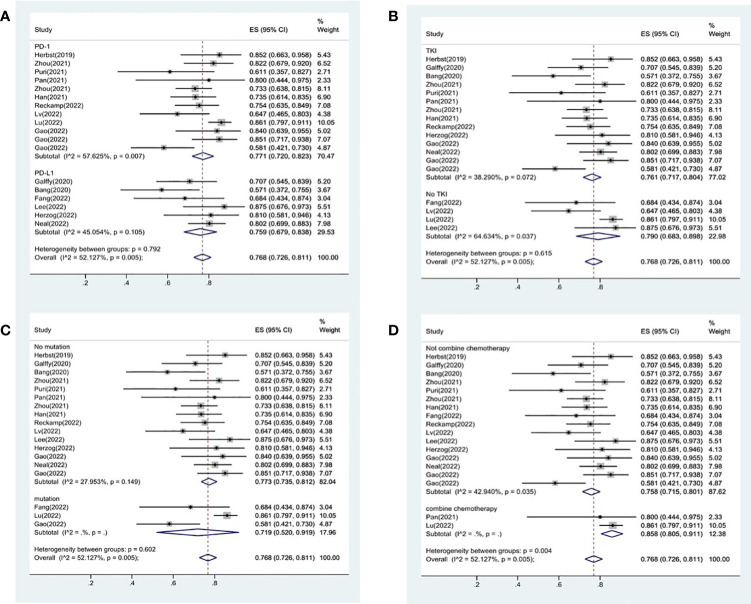
Subgroup analysis of DCR in immunotherapy inhibitor **(A)**, anti-angiogenic agent **(B)**, EGFR mutation **(C)** and whether or not to combine chemotherapy **(D)**.

18 studies included available data on DCR, and the DCR across the studies varied from 58 to 87%. The analysis showed a pooled DCR of 76.8% (95% CI: 72.6%–81.1%) and revealed considerable heterogeneity (I^2 =^ 52.1%, P=0.005, **Figure 2B**). As the significant heterogeneity of DCR across the studies existed, we also further investigated potential sources of heterogeneity by meta-regression and subgroup analysis. For the meta-regression analysis, we also selected the same 9 variables (year, region, phase, immunotherapy inhibitor, anti-angiogenic drug, tumor histology, study design, EGFR mutation and whether or not to combine chemotherapy). The results showed the phase of study contributed to the heterogeneity of DCR (**Figure 3B**), thus we carried out further subgroup analysis of the phase of the study, the results show a high DCR for Phase III studies, but with only one Phase III study, more studies need to be included in the analysis before certain conclusions can be drawn (**Figure S2**). We also chose four factors (immunotherapy inhibitor, anti-angiogenic agent, EGFR mutation, whether or not to combine chemotherapy) for further subgroup analysis. Subgroup analysis revealed that the pooled DCR in patients who received PD-1 as immunotherapy was 77.1% (95% CI: 72.0%–82.3%).The pooled DCR of patients receiving TKI as treatment was 76.1% (95% CI: 71.7%–80.4%). The pooled DCR of patients without EGFR mutation was 77.3% (95% CI: 73.5%–81.2%). The pooled DCR of patients receiving chemotherapy was 85.8% (95% CI: 80.5%–91.1%), which was higher than that of patients who did not receive chemotherapy, with a statistical difference (**Figure 5**).

### Efficacy evaluation of OS and PFS

**Figure 6:**
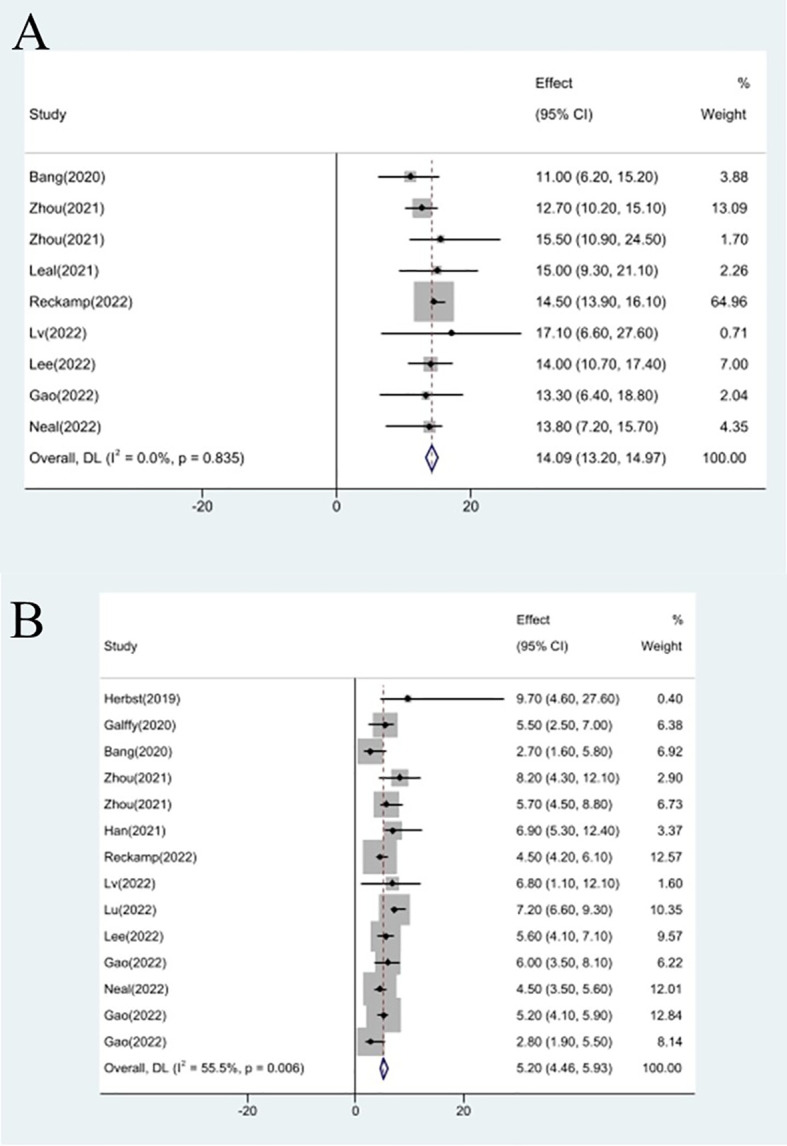
Forest plot about the pooled results of OS **(A)** and PFS **(B)**.

**Figure 7:**
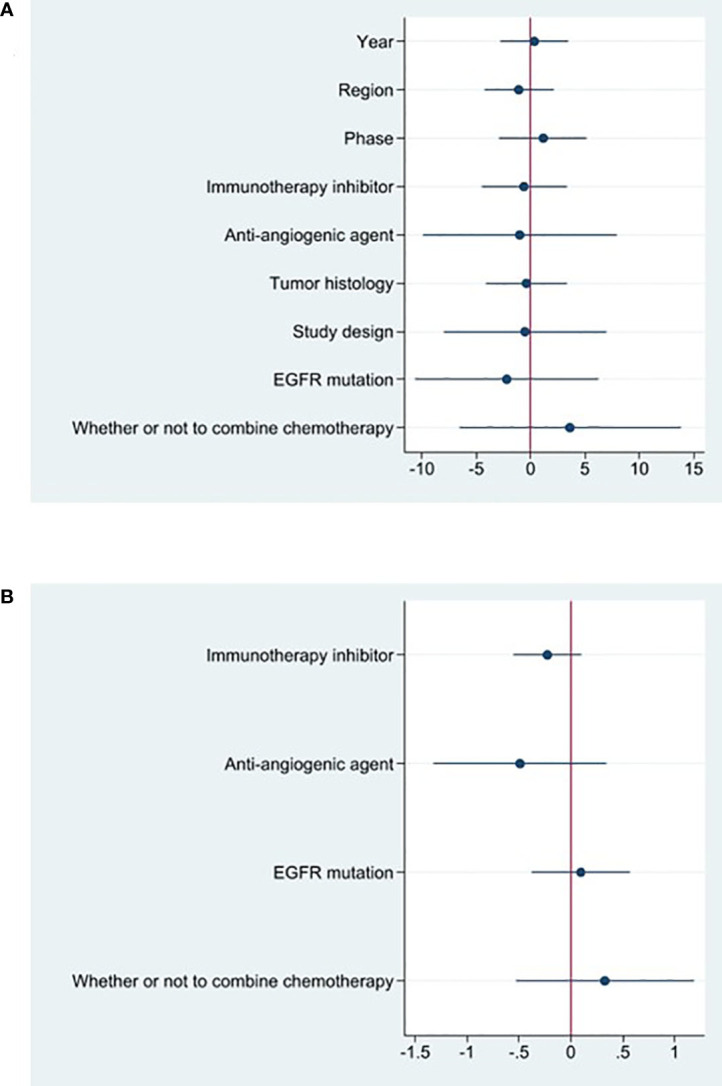
Coefplot of the results of meta-regression of PFS **(A)** and AE **(B)**.

**Figure 8:**
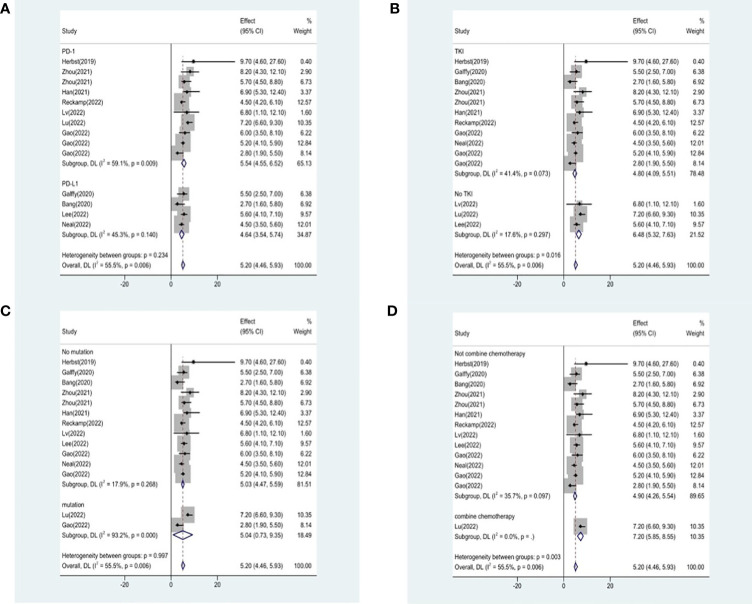
Subgroup analysis of PFS in immunotherapy inhibitor **(A)**, anti-angiogenic agent **(B)**, EGFR mutation **(C)** and whether or not to combine chemotherapy **(D)**.

Studies that did not provide specified 95% confidence intervals were eliminated, 9 studies included in the analysis reported OS and 14 studies reported PFS. In the random-effects model, the pooled median OS was 14.09 months (95% CI 13.20–14.97 months), as shown in **Figure 6A**. With regard to PFS, the results showed that the pooled median PFS was 5.20 months (95% CI: 4.46–5.93 months, **Figure 6B**). We further analyzed potential sources of heterogeneity by using meta-regression and subgroup analysis because there was a heterogeneity of PFS between different trials(I^2 =^ 55.5%, P=0.006). For the meta-regression analysis, we also selected the same 9 variables (year, region, phase, immunotherapy inhibitor, anti-angiogenic drug, tumor histology, study design, EGFR mutation, whether or not to combine chemotherapy). According to the meta-regression results, there is no statistically significant difference between the p-values for each variable (**Figure 7A**). We also chose four factors (immunotherapy inhibitor, anti-angiogenic agent, EGFR mutation and whether or not to combine chemotherapy) for further subgroup analysis. Subgroup analysis revealed that the pooled median PFS in patients who received PD-1 as immunotherapy was 5.54 months (95% CI: 4.55–6.52).The pooled median PFS of patients receiving TKI as treatment was 4.80 months (95% CI:4.09–5.51), which was shorter than that of patients who received recombinant human endostatin and bevacizumab as treatment, with a statistical difference. The pooled median PFS of patients without EGFR mutation was 5.03 months (95% CI: 4.47–5.59). The pooled median PFS of patients receiving chemotherapy was 7.20 months (95% CI: 5.85–8.85), which was higher than that of patients who did not receive chemotherapy, with a statistical difference (**Figure 8**).

### Toxicities

**Figure 9:**
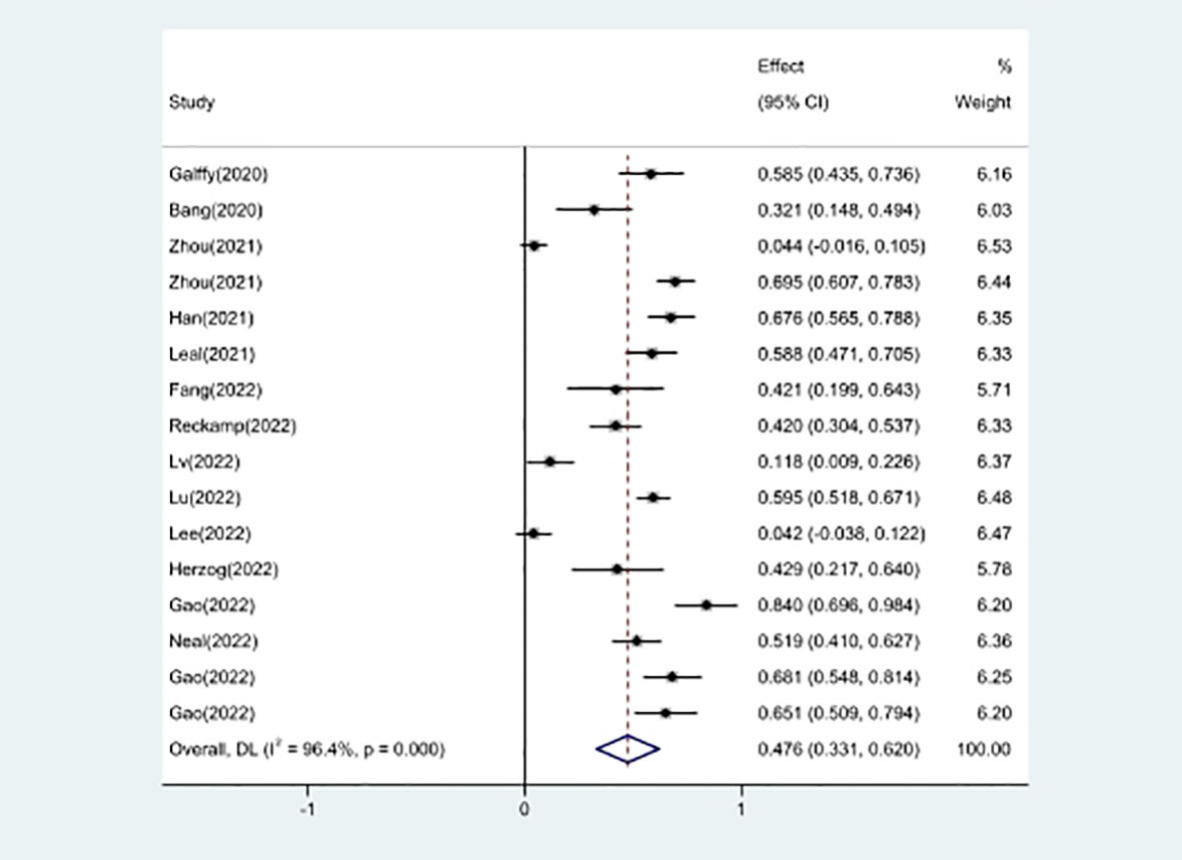
Forest plot about the pooled results of AE.

**Figure 10:**
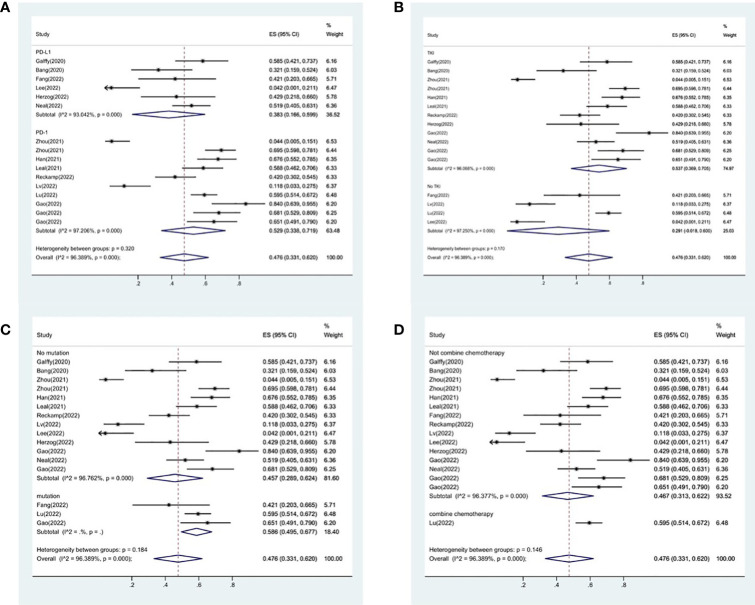
Subgroup analysis of AE in immunotherapy inhibitor **(A)**, anti-angiogenic agent **(B)**, EGFR mutation **(C)** and whether or not to combine chemotherapy **(D)**.

16 studies included in the meta-analysis provided the available incidence of AE (≥ grade 3). The most commonly reported adverse event was hypertension, the pooled AE≥ grade 3 was 47.6% (95% CI 33.1%–62.0%), as shown in **Figure 9**. As the significant heterogeneity of AE≥ grade 3 across the studies existed, we also further investigated potential sources of heterogeneity by meta-regression and subgroup analysis. For the meta-regression analysis, we selected the same 4 variables (immunotherapy inhibitor, anti-angiogenic drug, EGFR mutation, whether or not to combine chemotherapy), there is no statistically significant difference between the p-values for each variable (**Figure 7B**). We also chose the same four factors (immunotherapy inhibitor, anti-angiogenic agent, EGFR mutation and whether or not to combine chemotherapy) for further subgroup analysis. Subgroup analysis revealed that the pooled AE≥ grade 3 in patients who received PD-1 as immunotherapy was 52.9% (95% CI: 33.8%–71.9%). The pooled AE≥ grade 3 of patients receiving TKI as treatment was 53.7% (95% CI: 36.9%–70.5%). The pooled AE≥ grade 3 of patients without EGFR mutation was 45.7% (95% CI: 28.9%–62.4%). The pooled AE≥ grade 3 of patients receiving chemotherapy was 59.5% (95% CI: 51.4%–67.2%, **Figure 10**).

### Sensitivity analysis

One study was left out at a time throughout the sensitivity analysis to determine how it might affect the combined results. According to the analysis’s findings, no one research significantly affected any of the pooled results with 95% CIs. This proved the overall reliability of the meta-analysis’s findings. **Figure S3** displays the sensitivity analysis’s findings.

### Publication bias

Using Egger’s tests, the publication bias was estimated. We assumed that no publication bias existed for the ORR (Egger’s test: 0.929), the PFS (Egger’s test: 0.321), the OS (Egger’s test: 0.559), and the AE grade 3 (Egger’s test: 0.067). The test findings were consistent with most of the results, except for the DCR (Egger’s test: 0.016). The Funnel graphs of publication bias were showed in **Figure S4**.

## Discussion

In this era of numerous drugs, the treatment of lung cancer has evolved fast. The availability of second or later-line treatment for advanced non-small cell lung cancer is still constrained. Docetaxel was established as the standard chemotherapy regimen for second-line treatment of NSCLC in the TAX317/TAX320 studies (37, 38) and the results of the TAX317 study demonstrated that it significantly increased overall survival when used in the treatment of driver-negative advanced NSCLC compared to best supportive care (7 months vs. 4.6 months). The advent of immunotherapeutic medications has further changed the paradigm of second or later-line therapy for patients with advanced NSCLC. Based on the results of KEYNOTE-010, CheckMate 078 and OAK (9, 39, 40). PD-1/PD-L1 inhibitors monotherapy have been approved by the Food and Drug Administration (FDA) and the National Medical Products Administration (NMPA) of China for the second or later-line treatment of patients with driver-negative advanced non-small cell lung cancer. Alfredo Tartarone also conducted a Meta-analysis (41), this meta-analysis confirms the superiority of ICIs over docetaxel in pretreated non-small-cell lung cancer patients and would indicate a slight benefit from anti-PD-1 than from anti-PD-L1 inhibitors. The ALTER0303 study (12) demonstrated a median OS extension of 3.3 months for patients in the anlotinib arm compared to the placebo arm (9.6 months vs. 6.3 months); and a median PFS extension of 4.0 months (5.4 months vs. 1.4 months). Based on the results of this study, anlotinib was approved for third-line treatment of patients with driver-negative advanced non-small cell lung cancer. There is a dearth of scientific data to support the use of combination regimens in the second or later-line therapy of patients with advanced non-small cell lung cancer, hence several clinical trials are being carried out globally to further investigate the viability of combination regimens.

Combination regimen based on small molecule VEGF signaling pathway inhibitors like anlotinib, apatinib, and lenvatinib is a popular issue in current research. Many academics have provided compelling justifications for the mechanisms at work when small molecule VEGF signaling pathway inhibitors are taken with additional medications, of these, the treatment options with the most established grounded theory were PD-1/PD-L1 inhibitors plus anti-angiogenic agents. A blockade of the VEGF signaling pathway with anti-angiogenic agents can have an enhanced anti-tumor immune effect because prior research has demonstrated that the Vascular Endothelial Growth Factors (VEGF)/VEGFR signaling pathway inhibits anti-tumor immune responses not only by producing a hypoxic microenvironment but also through other complex mechanisms to produce immunosuppressive effects (42–45). Activated immune cells can inhibit tumor angiogenesis both directly and indirectly, according to studies (46–48), resulting in a positive feedback loop between immunotherapy and anti-angiogenic treatment. A growing number of clinical studies focus on PD-1/PD-L1 inhibitors plus anti-angiogenic agents.

In our meta-analysis, nineteen clinical trials with 931 patients were included to evaluate the efficacy and safety of PD-1/PD-L1 inhibitors plus anti-angiogenic agents as second or later-line treatment for patients with advanced non-small cell lung cancer. The pooled analyses presented that PD-1/PD-L1 inhibitors plus anti-angiogenic agents exhibited efficacy and manageable safety with promising ORR, DCR, OS, and PFS. The pooled results showed that the ORR and DCR were 22.4% and 76.8%, respectively, and the median OS and PFS were 14.09 months and 5.20 months, respectively. The subgroup analysis indicated that it was likely that combination of chemotherapy resulted in an increased ORR and DCR. The pooled median PFS of patients receiving small molecule VEGF signaling pathway inhibitors as treatment was lower than that of patients who received recombinant human endostatin and bevacizumab as treatment. In the meantime, our research shows that the pooled AE≥ grade 3 was 47.6%, and the most commonly reported adverse event was hypertension. Xiaoying Sun et al. conducted a meta-analysis (49) to assess the immune-related adverse events associated with programmed cell death protein-1 and programmed cell death ligand 1 inhibitors for non-small cell lung cancer, study showed only a 4% probability of serious adverse events. However, that study included data from a large number of patients and addressed adverse reactions associated with PD-1 and PD-L1 drugs, whereas our study addressed a smaller number of patients in the study and addressed data from a combination anti-vascular drug regimen. And a large number of in our meta-analysis have shown that serious adverse events are hypertensive, and associated with anti-vascular drugs, the adverse events are relatively safe and manageable.

In monotherapy, nivolumab’s effectiveness and safety in treating advanced non-small-cell lung cancer after prior treatment were evaluated by a meta-analysis (50). According to the research, the pooled ORR of the 817 patients who received nivolumab was 20%, and the pooled DCR of the 657 patients who received nivolumab was 36%. According to OAK (9) data, the ORR of 425 patients in the atezolizumab group was 14%, while the DCR was 49%. According to ALTER 0303’s results (12), the ORR of 437 patients in the anlotinib group was 9.2%, and the DCR was 81%. The ORR of 353 patients in the nintedanib group was 9.1%, and the DCR was 60.9%, according to the results of LUME-Lung 2 (51). And the data from our study suggest that PD-1/PD-L1 inhibitors plus anti-angiogenic agents have more promising efficacy as second or later-line treatment in patients with advanced non-small cell lung cancer.

Another hot topic in current research is the use of chemotherapy plus PD-1/PD-L1 inhibitors combination regimens as second or later-line treatment for patients with advanced non-small cell lung cancer. The effectiveness of nivolumab with docetaxel in the second or later-line therapy of patients with advanced non-small cell lung cancer was examined in a clinical study (52). According to the results of the trial, the ORR was 41.8% and the DCR was 80% in the nivolumab plus docetaxel group. As second-line treatment for advanced non-small cell lung cancer without targetable mutations, sintilimab with docetaxel showed promising outcomes, according to Zhang et al. (53). The ORR was 36.7%, the DCR was 76.6%, the median PFS was 5.0 months, and the median OS was 13.4 months. The effectiveness of these two trials’ findings was comparable to that of PD-1/PD-L1 inhibitors combined with anti-angiogenic medicines in this meta-analysis. Real-world data from our hospitals were analyzed by our team, and the findings of the study demonstrated that PD-1/PD-L1 inhibitors plus anti-angiogenic medicines were superior than PD-1/PD-L1 inhibitors plus chemotherapy (54). According to the data indicated above, PD-1/PD-L1 inhibitors combined with anti-angiogenic drugs had a satisfactory impact when used as a second or later-line treatment for patients with advanced non-small cell lung cancer. Our study is based on data from second and later line therapy, a population for which most existing clinical studies have not focused on PD-L1 expression in a timely manner, and which is therefore not mentioned in the extensive literature. Even the available studies agree that different countries and different populations will have different PD-L1 expressions, but it is unknown whether this difference causes differences in therapeutic efficacy. This also suggests that studying the relationship between PD-L1 expression and drug efficacy after treatment with various drugs is a key research direction for the future.

In conclusion, our study has a number of advantages: First and foremost, our meta-analysis was conducted on better quality clinical trials, and a sufficient number of clinical trials were included. Second, we carry out rigorous statistical analysis of the data to ensure the stability and reliability of the results. The last but not least, we compared the results of previous studies to confirm the effectiveness of the treatment options. The meta-analysis’s findings are valuable for physicians in that they may be used to create more effective treatment strategies for various individuals in a clinical environment.

The present meta-analysis had some shortcomings. First, the included studies showed significant heterogeneity. Despite our best efforts, we were unable to accurately identify the source of heterogeneity using meta-regression and subgroup analysis. Second, because all of the included studies had small sample sizes and were noncontrolled trials, we were only able to assess the effectiveness and risk without drawing any firm conclusions. Third, we haven’t been able to thoroughly analyze the AE in further depth. In order to validate the clinical function of PD-1/PD-L1 inhibitors in combination with anti-angiogenic medicines in contrast to other medications and the general population, further large-scale RCTs need be developed.

## Conclusion

PD-1/PD-L1 inhibitors plus anti-angiogenic agents has promising efficacy and safety as second or later-line treatment in patients with advanced non-small cell lung cancer.

## Data availability statement

The original contributions presented in the study are included in the article/**Supplementary Material**. Further inquiries can be directed to the corresponding authors.

## Author contributions

Material preparation, data collection, analysis and writing were performed by SC and WM. WJ and SZ given some constructive discussions make revisions to the paper. QY and HG conceptualized the research process and guided its conduct. All authors contributed to the article and approved the submitted version.

## Supplementary material

Supplementary Figure 1 Risk of bias assessments for three randomized studies.

Supplementary Figure 2 Subgroup analysis of the phase of DCR.

Supplementary Figure 3 Sensitivity analysis. Sensitivity analysis for ORR **(A)**; Sensitivity analysis for DCR **(B)**; Sensitivity analysis for OS **(C)**; Sensitivity analysis for PFS **(D)**; Sensitivity analysis for AE **(E)**.

Supplementary Figure 4 The Funnel graphs of publication bias of ORR **(A)**, DCR **(B)**, OS **(C)**, PFS **(D)** and AE **(E)**.
