# Supplementary figures and images for: The benefit and risk of PD-1/PD-L1 inhibitors plus anti-angiogenic agents as second or later-line treatment for patients with advanced non-small-cell lung cancer: a systematic review and single-arm meta-analysis of prospective clinical trials
Source: Front Immunol. 2023 Aug 8;14:1218258. doi: 10.3389/fimmu.2023.1218258 (PMC10442655; doi:10.3389/fimmu.2023.1218258)

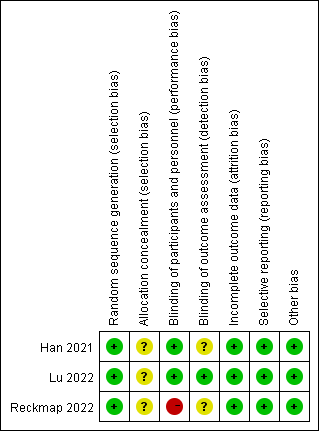

Supplement: Supplementary Figure 1 — Risk of bias assessments for three randomized studies. [file Image_1.png]

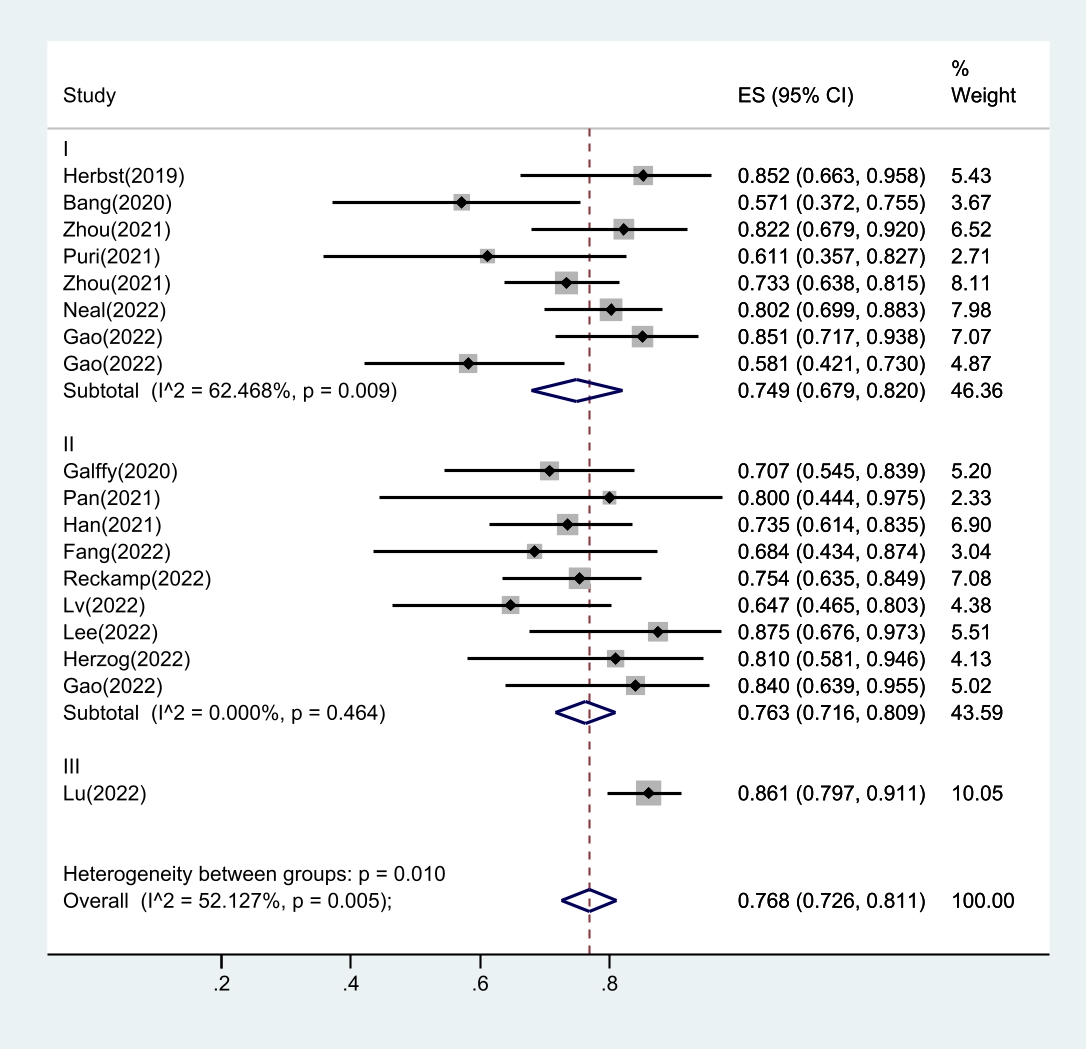

Supplement: Supplementary Figure 2 — Subgroup analysis of the phase of DCR. [file Image_2.jpeg]

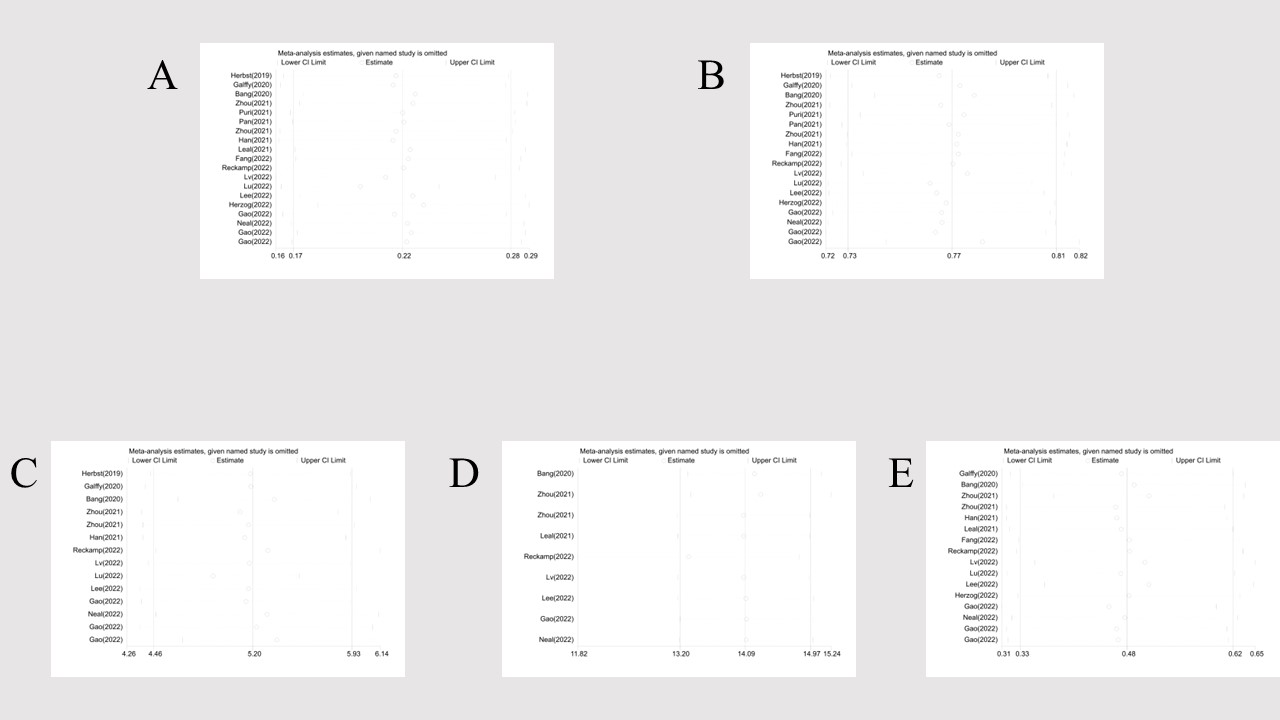

Supplement: Supplementary Figure 3 — Sensitivity analysis. Sensitivity analysis for ORR (A); Sensitivity analysis for DCR (B); Sensitivity analysis for OS (C); Sensitivity analysis for PFS (D); Sensitivity analysis for AE (E). [file Image_3.jpeg]

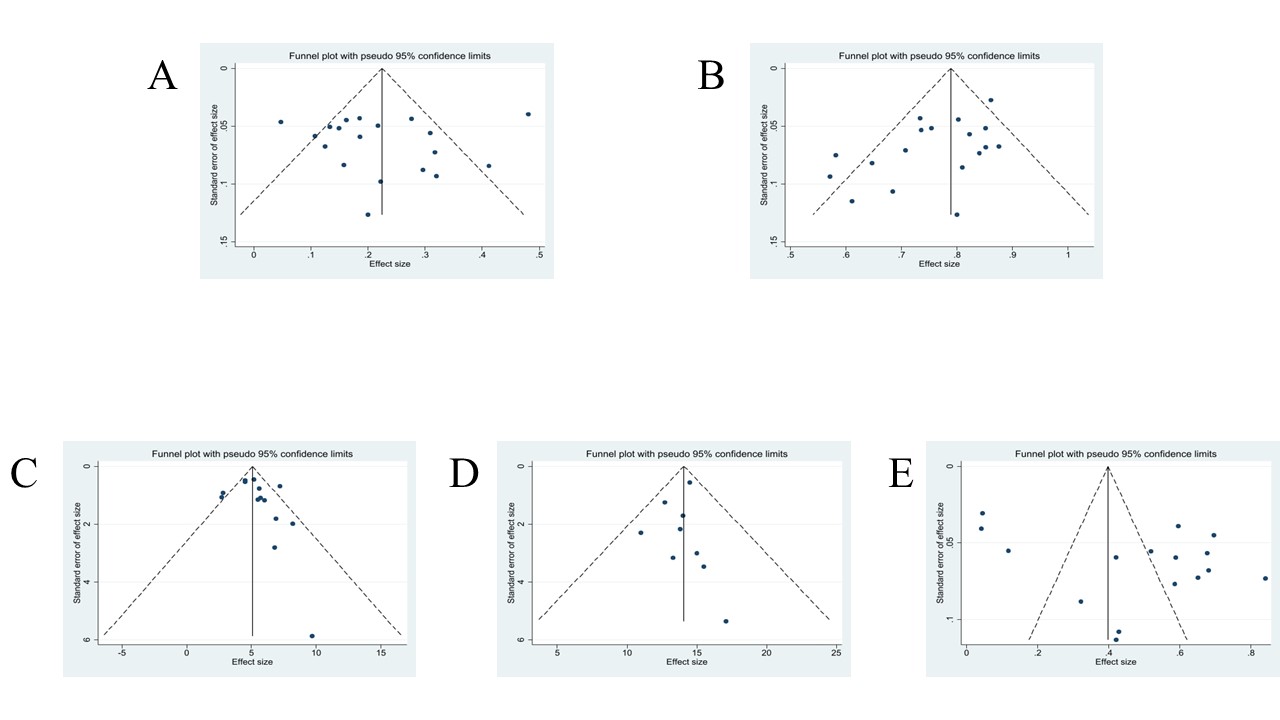

Supplement: Supplementary Figure 4 — The Funnel graphs of publication bias of ORR (A), DCR (B), OS (C), PFS (D) and AE (E). [file Image_4.jpeg]
