# Supplementary material for: The benefit and risk of PD-1/PD-L1 inhibitors plus anti-angiogenic agents as second or later-line treatment for patients with advanced non-small-cell lung cancer: a systematic review and single-arm meta-analysis of prospective clinical trials
Source: Front Immunol. 2023 Aug 8;14:1218258. doi: 10.3389/fimmu.2023.1218258 (PMC10442655; doi:10.3389/fimmu.2023.1218258)
Supplement: Supplementary file 5 [file Table_1.docx]

**Table S1.** Search strategy

((("Carcinoma, Non-Small-Cell Lung"[Mesh]) OR (((((((((((Carcinoma, Non Small Cell Lung[Title/Abstract]) OR (Carcinomas, Non-Small-Cell Lung[Title/Abstract])) OR (Lung Carcinoma, Non-Small-Cell[Title/Abstract])) OR (Lung Carcinomas, Non-Small-Cell[Title/Abstract])) OR (Non-Small-Cell Lung Carcinomas[Title/Abstract])) OR (Non-Small-Cell Lung Carcinoma[Title/Abstract])) OR (Non Small Cell Lung Carcinoma[Title/Abstract])) OR (Carcinoma, Non-Small Cell Lung[Title/Abstract])) OR (Non-Small Cell Lung Carcinoma[Title/Abstract])) OR (Non-Small Cell Lung Cancer[Title/Abstract])) OR (Nonsmall Cell Lung Cancer[Title/Abstract]))) AND (("Programmed Cell Death 1 Receptor"[Mesh]) OR ((((((((((((((PD-1[Title/Abstract]) OR (PD-L1[Title/Abstract])) OR (Programmed Death Ligand 1 Inhibitors[Title/Abstract])) OR (Programmed Cell Death Protein 1 Inhibitor[Title/Abstract])) OR (Pembrolizumab[Title/Abstract])) OR (Nivolumab[Title/Abstract])) OR (Atezolizumab[Title/Abstract])) OR (Durvalumab[Title/Abstract])) OR (Camrelizumab[Title/Abstract])) OR (Tislelizumab[Title/Abstract])) OR (Sintilimab[Title/Abstract])) OR (Toripalimab[Title/Abstract])) OR (Sugemalimab[Title/Abstract])) OR (Serplulimab[Title/Abstract])))) AND (("Angiogenesis Inhibitors"[Mesh]) OR ((((((((((((((((Angiogenetic Antagonist[Title/Abstract]) OR (Angiostatic Agent[Title/Abstract])) OR (Anti-Angiogenetic Agent[Title/Abstract])) OR (Anti Angiogenic Drug[Title/Abstract])) OR (Bevacizumab[Title/Abstract])) OR (Ramucirumab[Title/Abstract])) OR (Nintedanib[Title/Abstract])) OR (Anlotinib[Title/Abstract])) OR (Apatinib[Title/Abstract])) OR (Vandetanib[Title/Abstract])) OR (Sunitinib[Title/Abstract])) OR (Pazopanib[Title/Abstract])) OR (Lenvatinib[Title/Abstract])) OR (Cediranib[Title/Abstract])) OR (Motesanib[Title/Abstract])) OR (Axitinib[Title/Abstract])))
